# Changes in concentration of *Alternaria* and *Cladosporium* spores during summer storms

**DOI:** 10.1007/s00484-012-0604-0

**Published:** 2012-11-19

**Authors:** Agnieszka Grinn-Gofroń, Agnieszka Strzelczak

**Affiliations:** 1 Department of Plant Taxonomy and Phytogeography, University of Szczecin, Wąska 13 Street, 71-415 Szczecin, Poland; 2 Faculty of Food Sciences and Fisheries, West Pomeranian University of Technology, Papieża Pawła VI 3 Street, 71-459 Szczecin, Poland

**Keywords:** Fungal spores, Ozone, Thunderstorms, Artificial neural networks, *Alternaria*, *Cladosporium*

## Abstract

Fungal spores are known to cause allergic sensitization. Recent studies reported a strong association between asthma symptoms and thunderstorms that could be explained by an increase in airborne fungal spore concentrations. Just before and during thunderstorms the values of meteorological parameters rapidly change. Therefore, the goal of this study was to create a predictive model for hourly concentrations of atmospheric *Alternaria* and *Cladosporium* spores on days with summer storms in Szczecin (Poland) based on meteorological conditions. For this study we have chosen all days of June, July and August (2004–2009) with convective thunderstorms. There were statistically significant relationships between spore concentration and meteorological parameters: positive for air temperature and ozone content while negative for relative humidity. In general, before a thunderstorm, air temperature and ozone concentration increased, which was accompanied by a considerable increase in spore concentration. During and after a storm, relative humidity increased while both air temperature ozone concentration along with spore concentrations decreased. Artificial neural networks (ANN) were used to assess forecasting possibilities. Good performance of ANN models in this study suggest that it is possible to predict spore concentrations from meteorological variables 2 h in advance and, thus, warn people with spore-related asthma symptoms about the increasing abundance of airborne fungi on days with storms.

## Introduction

The number of days with thunderstorms in Poland depends on the region, and varies between 15 days in the north-western part of the country and 33 days in the south-eastern part. The number in any individual region may change considerably from one year to another. This is typical for the transitional climate of Poland. A similar trend of increasing occurrence of summer thunderstorms from the northwest to the southeast was observed in the Czech Republic and in Germany (Kożuchowski 1998).

The highest concentrations of *Alternaria* and *Cladosporium* spores occur during summer from June to September in many parts of Europe, e.g. Italy (Ballero et al. 1992), Sweden (Hjelmroos 1993), Denmark (Larsen and Gravesen 1991) and in many regions of Poland, e.g. Lublin (Konopińska 2004), Kraków, Poznań, Warszawa, Ostrowiec Świętokrzyski and Zakopane (Stępalska et al. 1999).

The fungal spores of *Alternaria* and *Cladosporium* species are known to cause allergic sensitization and seasonal asthma symptoms. Most asthma cases were recorded during the hot and dry summer days, however there is some evidence for the occurrence of asthma symptoms during thunderstorms. The asthma epidemics have been reported in association with thunderstorms in Birmingham, Nottingham and London, United Kingdom in 1984, 1994 as well as in Melbourne and Wagga Wagga, Australia in 1984, 1987, 1989 and 1997 (Alderman et al. 1986; Bellomo et al. 1992; Egan 1985; Marks et al. 2001; Packe and Ayers 1986; Venables et al. 1997). A recent study from Canada reported a strong association between emergency visits for asthma and thunderstorms and suggested that the mechanism might be through an increase in fungal spore counts (Dales et al. 2003). Pulimood et al. (2007) performed a study of an epidemic of asthma admissions associated with thunderstorm outflow in the United Kingdom. Analysis of the atmosphere indicated that high levels of fungal spores (i.e. *Alternaria* and *Cladosporium*) were present and that *Alternaria* spores in the air were highly correlated with the admission rate for acute asthma. The authors conclude that *Alternaria* exposure is another important factor in thunderstorm-related asthma. Previous studies (Dales et al. 2003) have demonstrated increased airborne levels of fungal spores during thunderstorms, although a direct casual effect has not been established. Girgis et al. (2000) showed that 61 % of patients with thunderstorm-related asthma were sensitized to *Cladosporium* species.

Before and during thunderstorms, the values of meteorological parameters rapidly change. This may be the cause of asthma symptoms. Pulimood et al. (2007) suggest that the most important mechanisms associated with thunderstorms are fragmentation of *Alternaria* species spores into easily respirable allergenic fragments, a high concentration of fungal spores, and their transportation over large distances. Other associated factors, such as ozone and a sudden reduction in temperature with an increase in humidity, might contribute to bronchial hyperresponsiveness.

Therefore, the aim of this study was to predict hourly concentrations of *Alternaria* and *Cladosporium* spores, two of the most allergenic fungal species, based on changes in meteorological conditions on days with summer storms in Szczecin (Poland) and, thus, warn people with spore-related asthma symptoms about the increasing abundance of airborne fungi.

## Materials and methods

The data evaluated in this study were collected in Szczecin which is located in the north-west part of Poland. The annual mean temperature is around 8 °C and the difference between warmer and colder monthly averages is considerable. Mean, annual air humidity is 80 %, and the total annual mean precipitation varies between 500 mm and 550 mm. Average wind speed is 3.3 m/s and dominated by westerly and south-westerly winds. In Szczecin the average annual number of days with storms is 15 and most (about 10) occur in summer from June to August. In 2004–2009 the average number of storms in June was two, and four each in both July and in August (Kożuchowski and Degirmendžić 2005).

The study was done using the methods described by the British Aerobiology Federation (1995). The investigation was based on aerobiological monitoring performed in 2004–2009. Hourly counts of airborne fungal spores were performed using a 7-day recording Lanzoni volumetric trap. The trap was installed on a rooftop in Szczecin city, district Śródmieście, at a height of 21 m above ground level. Hourly records of the airborne fungal spores were obtained by counting all spores in one longitudinal transect at x 400 magnification using a light microscope MK 348 with phase contrast optics. The final counts of fungal spores were expressed as hourly numbers of spores per cubic meter of air. The meteorological data covering 6 years of studies were provided by the Automatic Weather Station (Vaisala MAWS101). The meteorological station was located in the immediate neighbourhood of the Lanzoni trap. The following hourly parameters were analysed: air temperature, relative humidity, air pressure and wind speed. Additionally, the concentration of ozone was included in this study because this gas occurs in the lower atmosphere during storms and is formed in the polluted air of large cities exposed to bright light. The values of ozone were monitored using special sensors (Thermo 49i: accuracy of 1.0 ppb, 0.5 ppb detection limit, zero drift of <1.0 ppb (24) mounted on the weather station).

For this study we chose 19 days in June, July and August (2004–2009) having particular types of thunderstorms (convective). This kind of storm is typical for the summer period and appears inside a homogeneous air mass. All storms occurred during hot weather in summer, generally in the afternoon. They are short, accompanied by extensive and rapid electrical discharges.

The total number of storms during the period analyzed was 60, of which 19 were convective. Days with thunderstorms included in our analysis were characterized by rainfall close to 0 mm, average air temperature above 20 °C, mean daily relative humidity above 70 % and occurrence of a storm with intense electrical discharges in the afternoon or at night. In 2004 the days with covective thunderstorm were four, with two in 2005, three in 2006, two in 2007, three in 2008 and five in 2009.

The final data set included 19 days of hourly data (24 h per day) which gave 456 cases. Statistical analyses were performed using StatSoft software Statistica 9.0 (StatSoft, Inc 2009; Lula 2000; Tadeusiewicz 1993, 2001). Normality of consecutive variables was tested with the Kolmogorov-Smirnov, Lilliefors and chi-square tests under *p* = 0.05. In turn, linearity of dependencies was assessed visually on scatter plots and the normality of residues from linear regression was checked. Due to non-normality of the variables and non-linearity of the relationships between spore contents and meteorological variables recorded on the same hour, and up to 5 h prior, the Spearman’s rank correlation analysis was used. Finally, artificial neural network (ANN) models were created in order to check the performance of predictions of spore concentration from meteorological conditions 1–5 h in advance. The ANN method was chosen because it allows analysis of data but makes no assumptions about the form of relationships between airborne mycoflora and explanatory factors. Moreover, artificial neural networks are applicable for complex ecological data with imbalance, non-linear relationships and high-order interactions. In this study multi layer perceptrons (MLP) were applied, which mathematically perform a stochastic approximation of multivariate functions (Osowski 1996; Carling 1992; Lek and Guegan 1999). The consecutive neural networks were designed and trained using Automated Network Search (ANS). This tool tries a given number of networks of complexity within a specified range and with different activation functions. Several thousand networks were tested with the number of hidden neurons from 3 to 50 and with various hidden and output activation functions (linear, logistic, hyperbolic tangent, negative exponential, sine). For training, the most recommended technique was applied—Broyden-Fletcher-Goldfarb-Shanno algorithm—and the error function used was sum of squares. For training, cases were divided using bootstrap method into three subsets:

- Training (Tr)—used for training a neural network (67 % of the cases);
- Verification (Ve)—used for verifying performance of a network during training (33 % of the cases);
- Testing (Te)—used for assessing predictability and accuracy of a neural model on data not presented during training and validation (cases remained after creating a training subset during bootstrap; combats overfitting).

Division of the data set into three subsets was problematic because we had fewer data (430 cases) than we would ideally desire even for a single subset. We got around this problem by resampling. Experiments can be conducted using different divisions of the available data into training, testing, and validation sets. There are a number of approaches to this subset, whereby one of them is bootstrap in which new training and verification sets are formed by sampling with replacements from the available data set. In sampling with replacement, cases are drawn at random from the data set, with equal probability, and any one case may be selected any number of times. Due to the sampling process, it is likely that some of the original cases will not be selected, and these are used to form a test set, whereas other cases will have been duplicated. Consequently, the design decisions (such as the best configuration of neural network to use) were based upon a number of experiments with different subset examples and, thus, the results were much more reliable.

Model quality of the best neural network was assessed with the Spearman’s rank correlation coefficient between experimental and predicted data, calculated separately for three subsets. Special emphasis was placed on sensitivity analysis. It creates a ranking of input variables and is based on calculations of the error, when a given input variable is removed from the model. The ratio of the error for the complete model to the one with an ignored variable is the basis of ordering variables according to their importance.

## Results

The average spore concentrations during the 3-month studies ranged between 300,000 s/m^3^ to 550,000 s/m^3^ for *Cladosporium* and 11,000 s/m^3^ to 25,000 s/m^3^ for *Alternaria*. The spore concentration for both spore types on thunderstorm days was always very high (above 50,000 s/m^3^ for *Cladosporium* and above 7,000 s/m^3^ for *Alternaria*).

**Fig. 1:**
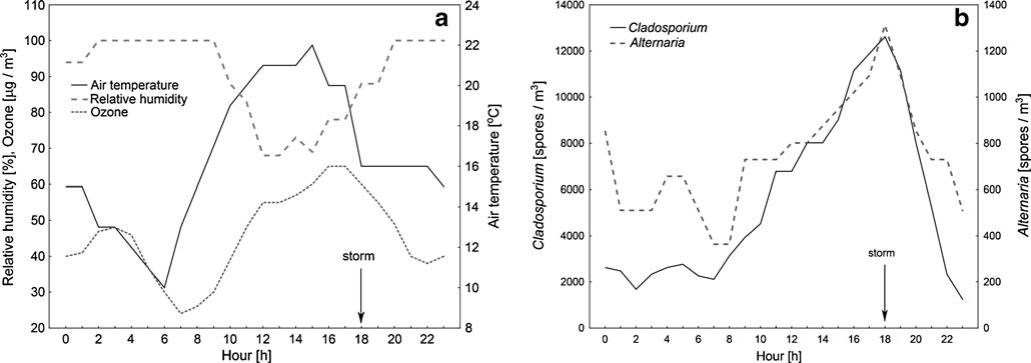
Changes in meteorological parameters (**a**) and *Cladosporium* and *Alternaria* hourly spore concentrations (**b**) recorded on 09 August 2006 (afternoon storm)

Preliminary data analysis revealed a clear pattern in meteorological parameters as well as in *Cladosporium* and *Alternaria* spore concentrations repeated independently of the time when a storm occurred, shown in the example of one day, 09 August 2006, in an afternoon storm (Fig. 1a,b). In general before a thunderstorm, air temperature and ozone concentration increased, which was accompanied by a considerable increase in spore concentration. During and after a storm, relative humidity increased and a decline in air temperature, ozone concentration and spore concentrations was observed. Wind speed and air pressure were relatively stable over a given day. In general, relationships between those two meteorological parameters and spore contents varied between consecutive days. Therefore, no clear pattern was observed and the resultant dependence was very weak.

**Fig. 2:**
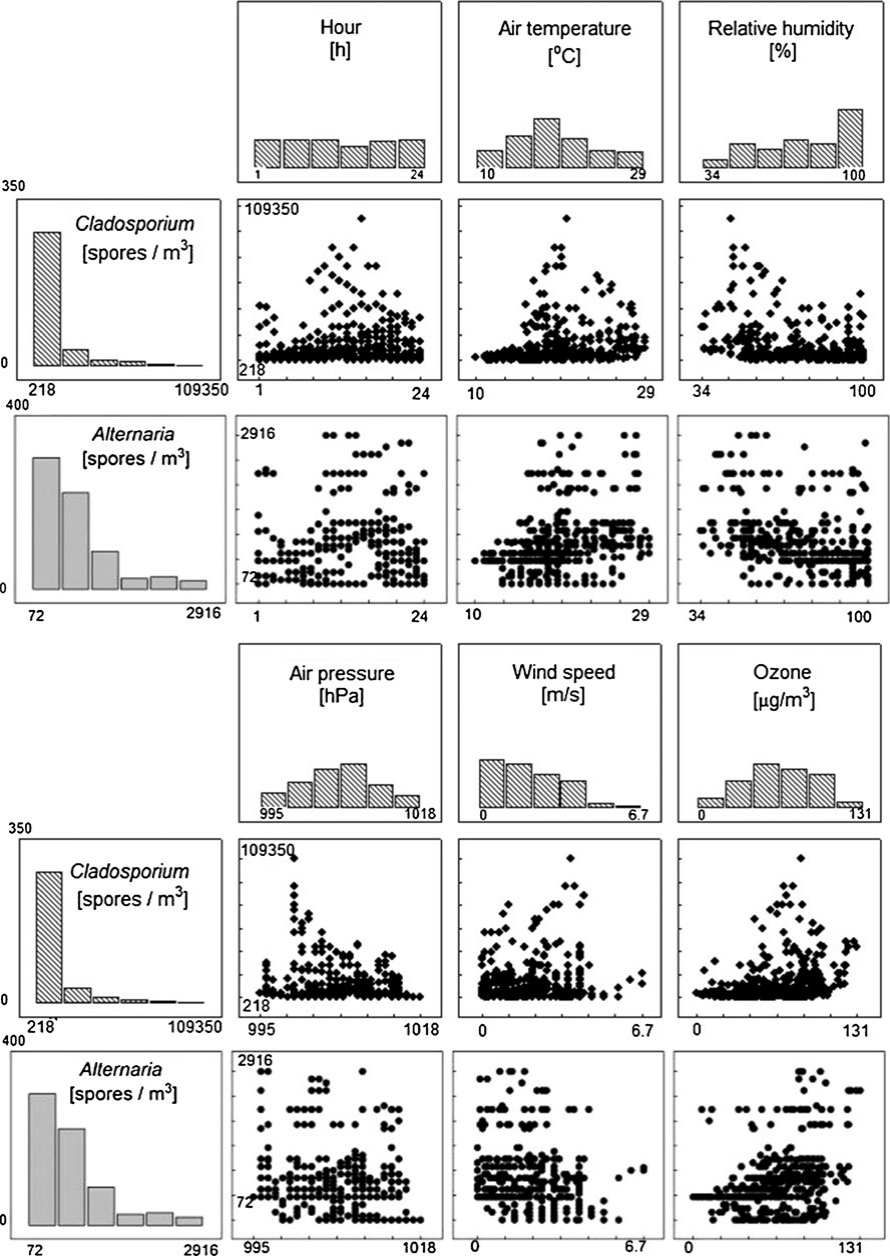
Frequency distributions and matrix scatter plots between *Cladosporium*, *Alternaria* spore concentrations, hour and meteorological factors

Figure 2 shows scatter plots between spore contents (y-axes, titles on the left of histograms) and consecutive meteorological conditions (x-axes, titles above histograms) as well as frequency distributions of variables on consecutive axes. Statistical analyses revealed non-normality (*p* < 0.05 for all variables and all normality tests) and non-linearity of the data set (*p* < 0.05 in all normality tests for all residues from linear regression).

Spearman’s rank correlation analyses indicated that *Cladosporium* spore concentration was significantly correlated with relative humidity (negative relationship), air temperature, and ozone (positive relationships). Considering the correlations with meteorological parameters recorded up to 5 h earlier, for relative humidity they were all significant and high with maximum correlation coefficient 2 h prior. In turn, for air temperature and ozone concentration, the correlations were similar up to 2 h prior and then decreased, particularly quickly for ozone content. Air pressure and wind speed did not seem to correspond with *Cladosporium* spore content. In the case of *Alternaria*, the relationships were similar but, additionally, air pressure and wind speed showed significant, although weak, negative correlations (Table 1). Those correlation analyses indicated that successful prediction can be achieved up to 2–3 h ahead.

**Table 1:** Spearman’s rank correlation coefficients between hourly *Cladosporium* and *Alternaria* spore concentration and meteorological variables recorded on the same hour and up to 5 h prior

Variable	Values over time					
Air temperature						
lag	0 h	−1 h	−2 h	−3 h	−4 h	−5 h
*Cladosporium*	0.41***	0.42***	0.39***	0.32***	0.23***	0.12*
*Alternaria*	0.40***	0.43***	0.41***	0.35***	0.25***	0.15**
Relative humidity						
lag	0 h	1 h	2 h	3 h	4 h	5 h
*Cladosporium*	−0.42***	−0.47***	−0.48***	−0.45***	−0.37***	−0.27***
*Alternaria*	−0.49***	−0.54***	−0.56***	−0.53***	−0.44***	−0.34***
Air pressure						
lag	0 h	1 h	2 h	3 h	4 h	5 h
*Cladosporium*	−0.07	−0.06	−0.06	−0.05	−0.05	−0.04
*Alternaria*	−0.12**	−0.13**	−0.13**	−0.13**	−0.13**	−0.12**
Wind speed						
lag	0 h	1 h	2 h	3 h	4 h	5 h
*Cladosporium*	0.06	0.08	0.09	0.10*	0.07	0.02
*Alternaria*	−0.13**	−0.09	−0.10*	−0.09	−0.09	−0.09
Ozone						
lag	0 h	1 h	2 h	3 h	4 h	5 h
*Cladosporium*	0.42***	0.39***	0.33***	0.24***	0.13*	0.03
*Alternaria*	0.35***	0.33***	0.27***	0.18***	0.05	−0.06

*p*-value: *p < 0.05. **p < 0.01, ***p < 0.001

The analysis of Spearman’s rank correlation coefficients between the explanatory variables revealed strong relationships in the case of relative humidity, air temperature and ozone concentration. That suggests redundancy of those parameters in explaining spore dynamics (Table 2).

**Table 2:** Spearman’s rank correlation coefficients between meteorological variables

Variable	Air temperature	Relative humidity	Air pressure	Wind speed	Ozone
Air temperature	1.00				
Relative humidity	−0.70***	1.00			
Air pressure	0.08	0.23***	1.00		
Wind speed	0.16**	−0.31***	−0.10*	1.00	
Ozone	0.67***	−0.60***	−0.11*	0.31***	1.00

*p*-value: *p < 0.05. **p < 0.01, ***p < 0.001

**Fig. 3:**
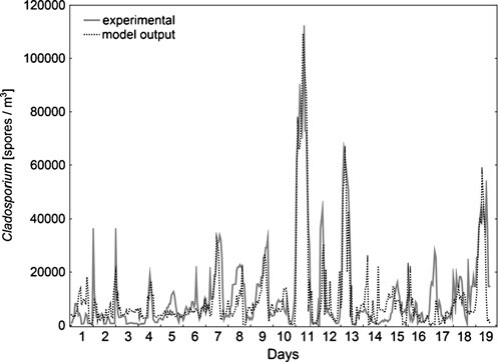
Comparison of *Cladosporium* spore concentrations obtained from measurements and those calculated from MLP 5-25-1 neural network

Finally, the predictive artificial neural network models were created. The best performance was attained with models forecasting spore concentrations from meteorological parameters 2 h ahead. For *Cladosporium* the best ANN model was a multi layer perceptron MLP 5-25-1 with five input neurons, 25 hidden neurons and one output neuron. Network performance (Spearman’s rank correlation coefficient between the experimental and calculated values) was at the level of 0.89, 0.80 and 0.79 for the training, testing and validation subsets, respectively. The model was trained with 66 epochs of Broyden-Fletcher-Goldfarb-Shanno algorithm. Sum of squares was applied as the error function. Hidden neurons had an exponential activation function while output neurons had logistic activation function. The obtained ANN model revealed good fit to experimental data, particularly for very high *Cladosporium* concentrations (Fig. 3). Sensitivity analysis showed that all the explanatory variables were important in the model. The best rank was reached by relative humidity and ozone. Air temperature, in spite of relatively high Spearman’s rank correlation coefficient, was at the last position (Table 3).

**Table 3:** Sensitivity analysis of predictive neural network models for *Cladosporium* (MLP 5-25-1) and *Alternaria* (MLP 5-20-1)

Concentrations		Relative humidity	Ozone	Wind speed	Air pressure	Air temperature
*Cladosporium*	Ratio	7.5	5.1	4.1	3.5	1.7
	Rank	1	2	3	4	5
*Alternaria*	Ratio	2.8	1.7	1.9	1.3	5.2
	Rank	2	4	3	5	1

**Fig. 4:**
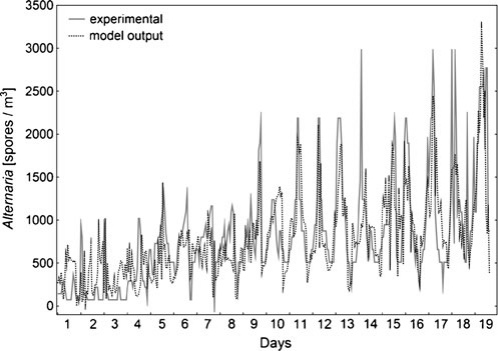
Comparison of *Alternaria* spore concentrations obtained from measurements and those calculated from MLP 5-20-1 neural network

The best ANN model for *Alternaria* was a multi layer perceptron MLP 5-20-1 with five input neurons, 20 hidden neurons and one output neuron. Network performance (Spearman’s rank correlation coefficient between the experimental and calculated values) was at the level of 0.78, 0.63 and 0.66 for the training, testing and validation subsets, respectively. The model was trained with 107 epochs of Broyden-Fletcher-Goldfarb-Shanno algorithm. Sum of squares was applied as the error function. Hidden and output neurons had exponential activation function. In spite of lower network performance, the ANN model revealed a similar fit to experimental data as that obtained for *Cladosporium* (Fig. 4). Sensitivity analyses revealed that all the variables were almost equally important in the model and the highest rank was reached by hour and air temperature (Table 3).

## Discussion

Pollen and spore forecasting has become an important aim in aerobiology. The objective is to provide accurate information on biological particles in the air to patients sensitized to airborne particles in order to help them manage their disease.

Many statistical techniques of data analysis are based on assumptions of linearity and normality that often cannot be fulfilled (Lek and Guegan 1999). The ANN technique can be applied to the problems that cannot be solved in any other effective way, and it is suited to predicting the concentration of airborne pollen or spores in relation to weather conditions.

Castellano-Méndez et al. (2005) created a neural model to predict the risk of level of *Betula* pollen in the air. Ranzi et al. (2003) used ANN to investigate the influence of meteorological conditions on the timing of the beginning of the pollen season and suggested that including the greatest possible number of parameters with a potential impact on the concentration of pollen in the air would produce reliable forecasts. Arizmendi et al. (1993) described a neural model which was able to predict near future values of pollen concentration. The serious drawback of their model was that the method could be used only after the beginning of the season and therefore was unable to predict the start of the pollen season.

The neural network model of the relationship between *Betula* pollen and meteorological factors created in Poland (Puc 2012) eliminates this drawback and can be used at any time to provide a forecast for the entire season using only meteorological variables.

Sánchez-Mesa et al. (2005) created a neural network model to forecast the severity of the Poaceae pollen season by using weather parameters prior to the pollen season. Their model has a high applicability in the area of prevention based on a series of meteorological values accumulated prior to the pollen season.

All these models predict concentration for a given type of pollen using standard meteorological variables. The specific weather conditions, even if they occurred, can be generalized in a model containing years of data. Hence it is important to create forecasts for particular types of weather, for example, a specific type of storm.

On the basis of the Bielec-Bąkowska (2003) investigations, in Szczecin there are on average 15 days during a year with thunderstorms. In comparison to other regions in Poland this is the smallest number but the variability in number of days is the largest. During the six-year period studied, the average number of days with thunderstorms in Szczecin was ten per year and days with particular consecutive thunderstorm were three per year.

Before and during a thunderstorm, the values of some meteorological parameters such as air temperature, relative humidity, wind speed and ozone, can change rapidly. Ozone levels generally are higher in summer, as a result of sunlight. Anderson et al. (2001) reported that ozone tended to be higher on high temperature days and the highest levels were noted during thunderstorms in summer. The same authors also noted a positive relationship between asthma admissions and increasing ozone in summer. They found that both asthma cases and concentrations of airborne fungal spores increased during thunderstorms.

Some studies have shown that *Cladosporium* spore concentrations are higher during periods of warm temperature and dry air (Hirst 1953) while other investigations have suggested that *Cladosporium* release is somehow related to thunderstorms (Allit 2000). The same author noted that concentration of *Cladosporium* spores had been high throughout the day with thunderstorms, and conditions were evidently favourable for the spore production and release. In Derby, Lewis et al. (2000) observed that *Cladosporium* spore concentrations increased sharply with the occurrence of a thunderstorm. A similar effect was noted in the case of *Alternaria* concentrations during a storm in Sydney (Marks and Bush 2007). Very high levels of both taxa were observed in Tulsa where rainfall caused spikes in airborne spore concentrations (Burch and Levetin 2002) as well as in Ontario, where concentrations of *Cladosporium* and *Alternaria* during thunderstorms were markedly higher (48 % and 28 %, respectively) than on days without thunderstorms (Dales et al. 2003). While examining an asthma epidemic following an evening thunderstorm, Venables et al. (1997) identified increases in *Cladosporium* spore concentrations, and dry winds released spores in the downdraft gusts immediately before the storm. Marks and Bush (2007) suggest that thunderstorms may be an important trigger for *Alternaria-*related exacerbations of asthma. The mechanism still remains uncertain. According to Allit (2000) the life cycles and ecology of the fungi involved could help explain how spore release mechanisms might have been affected by the storm. Leach (1976), in his electrostatic theory of spore release, suggests that spores and other particles to which they attach are similarly charged, and when the connection between them weakens or breaks, they start to repeal one another electrically. This process can be affected by vibration, changes in relative humidity and the quality of light. Changes of these factors occur at the beginning of a storm, and there are of course electrical changes involved in thunderstorms as well.

In addition to the increased spore concentrations of *Alternaria* and *Cladosporium*, changes in concentrations of other spore types were also reported. Allit (2000) observed that during a storm on 24 July 1994, there was a small transient rise of spores of several smut species, the loose dry uredinospores of rust fungi and a dense band of translucent dust. The *Cladosporium* concentration was only slightly increased, and after the rain a modest increase in the concentration of ascospores of *Didymella*, *P. nigricans* and Diatrypaceae occurred. The same author noted that during a storm on 6 June 1996, there was transient rise in grass pollen, followed by the release of ascospores of *Lewia*. Allit (2000) summarizes that the dry spora before a storm is succeeded after the rain by a damp air spora of ascospores. Lewis et al. (2000), examining the effects of airborne particles, chemical pollutants and meteorological conditions on asthma admissions in Derbyshire UK noted that *Didymella* and *Cladosporium* spore counts increased sharply with the occurrence of a thunderstorm, whereas grass pollen tended to be slightly reduced on the day of a thunderstorm, peaking in the days prior to the storm.

The analysis of relationships between the average hourly fungal spore concentrations and certain weather variables demonstrates that before a thunderstorm air temperature and ozone concentration increased and it was accompanied by an increase in spore concentrations. During and after a storm, relative humidity increased while air temperature, ozone concentration and spore content decreased. No clear pattern was observed for wind speed and air pressure and they correlated with *Alternaria* spore content significantly but very weakly. The first parameter probably changed significantly in time intervals shorter than 1 h, but the hourly average remained relatively stable. As for air pressure, this meteorological parameter reveals considerable consistency over 24 h and does not correspond with more dynamic changes in hourly spore concentrations.

Results obtained by Spearman’s rank correlation analyses with airborne spore concentrations (significant negative correlation for relative humidity and positive for ozone and air temperature) are similar to those obtained by Lewis et al. (2000) in Derby (United Kingdom). The authors monitored spore concentrations during thunderstorms over 3 years and used the same statistical method as we did for examining the relationships between spore counts and some meteorological parameters which occurred during thunderstorms. They observed positive correlations between spore concentrations, air temperature and ozone, but a negative relationship with relative humidity. The relationships with air temperature and relative humidity are well documented in aerobiological literature and their impact on spore concentrations in the air is well known (Hasnain 1993; Herrero et al. 1996; Hollins et al. 2004; Kurkela 1997; Munuera Giner and Carrión García 1995; Munuera Giner et al. 2001; Sabariego et al. 2004). The possible impact of ozone on concentrations of spores in the air is not so clear. The level of ozone in the air is related mainly to the air temperature. The Elminir (2005) study found that the average concentration values of ozone were the highest when temperatures were above 30 °C and showed a clear trend of increasing with increasing temperature. In high concentrations, ozone kills microorganisms by the oxidation of cellular components such as sulphydryl groups and amino acids peptides and proteins as well as through the oxidation of the cell membrane (Das et al. 2006). These processes can completely inhibit or effectively reduce the growth of fungi, e.g. *Cladosporium* sp. (Whangchai et al. 2006). On the other hand, Anderson et al. (2001) found an association between asthma admissions and ozone concentrations which can be explained by ionization of the air as a result of thunder activity. We have no evidence that a certain level of ozone stimulates the release of spores into the atmosphere but we also cannot exclude such a possibility.

Due to high correlations between relative humidity, air temperature or ozone concentration and spore concentrations, it is impossible to rank these variables according to the strength of relationships with spore concentration based on the Spearman’s rank correlation analysis. Conversely, ANN models, thanks to sensitivity analysis, revealed, that *Cladosporium* spore concentrations were primarily influenced by relative humidity and ozone concentration. For *Alternaria*, the role of ozone seemed to be of less importance. Moreover, ANN models, as an advanced statistical technique, revealed the role of factors which were not indicated as significant in correlation analysis, i.e. wind speed and air pressure. Similar results were obtained by Burch and Levetin (2002) at three sites in Oklahoma (Tulsa, Bixby and Hectorville). They used forward stepwise multiple regression analysis correlating log-transformed hourly spore concentrations with meteorological parameters. They found no relationship with wind speed but *Cladosporium* spore concentration was significantly correlated with air pressure and *Alternaria* concentration with relative humidity. Both spore types were also strongly influenced by air temperature.

The studies of Lin and Li (2000) showed a strong negative correlation between fungal spore concentration and wind speed when the wind speed was under 5 m/s. However, the fungal concentration increased as the wind speed was higher than 5 m/s. The authors took this to be evidence of the dilution effect of wind speed being overcome by more particles being raised in higher winds. Jones and Harrison (2004) concluded that maximum wind speed has to exceed a threshold speed to remove material from a surface by either blow off or movement of the surface. However at higher speed, spore concentrations may become diluted.

Packe and Ayers (1986) suggest that turbulent winds could increase the release of fungal spores or draw up sedimented fungal spores and resuspend them in the air. This is consistent with observations that spore concentrations approximately doubled on thunderstorm days.

The concentrations of *Cladosporium* and *Alternaria* spores observed during summer thunderstorms might be sufficient to cause sensitization even with only a limited exposure time (Burch and Levetin 2002). This can be important clinically, because sensitization to spore types found in high levels in this study can be linked to asthma severity. It is commonly accepted that the frequency of thunderstorms has recently increased rapidly both in Poland and in the whole of Europe (Bielec-Bąkowska 2003). Thunderstorm-related epidemics of asthma are alarming. Understanding these epidemics not only enhances knowledge about the mechanisms of severe asthma and the role of allergen exposure in its etiology, but may also help predict the events, prepare health authorities for their impact, and possibly even facilitate and provide health advice for at-risk individuals. In regions where thunderstorm-related epidemics are known to occur, health authorities should identify the specific conditions preceding the events and, when these conditions are met and a thunderstorm outflow is forecast, they should alert emergency departments and primary carers or doctors to prepare for the possible epidemic of exacerbated asthma (Marks and Bush 2007).

Good performance of the ANN models in this study suggests that it is possible to predict spore concentrations from meteorological variables 2 h in advance of a thunderstorm event. It could be very useful in real life situations for people with spore-related asthma symptoms. Usually forecasts of spore concentrations are more general and do not allow one to predict short-term variations in spore concentrations related to temporary atmospheric phenomena such as convective thunderstorms. An hourly predictive model would give the opportunity to warn sensitive individuals about the impending increase in risk.

Although this study was limited to one localized geographic area, the generalizability of our results is suggested by previous observations in other parts of world (Alderman et al. 1986; Bellomo et al. 1992; Egan 1985; Marks et al. 2001; Packe and Ayers 1986; Venables et al. 1997). Similarly designed studies should be replicated in other geographic areas to better assess the applicability in these areas.
